# Genetics and genomic medicine in Saudi Arabia

**DOI:** 10.1002/mgg3.97

**Published:** 2014-07-30

**Authors:** Fowzan S Alkuraya

**Affiliations:** 1 Department of Genetics, King Faisal Specialist Hospital and Research CenterRiyadh, Saudi Arabia; 2 Department of Anatomy and Cell Biology, College of Medicine, Alfaisal UniversityRiyadh, Saudi Arabia


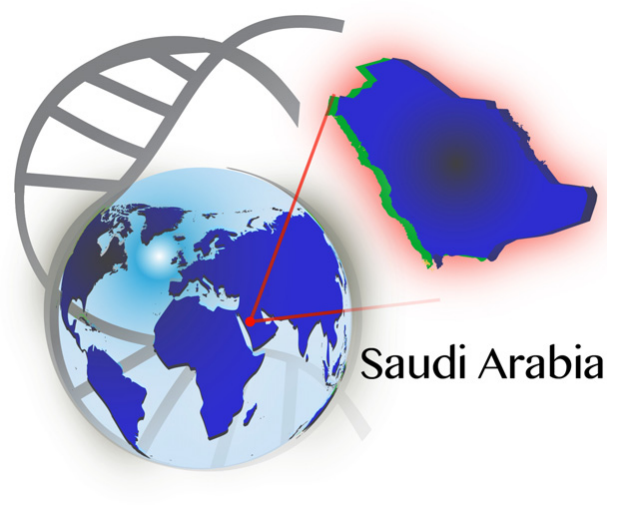


## Saudi Arabia: The New Arabia

**Figure 1:**
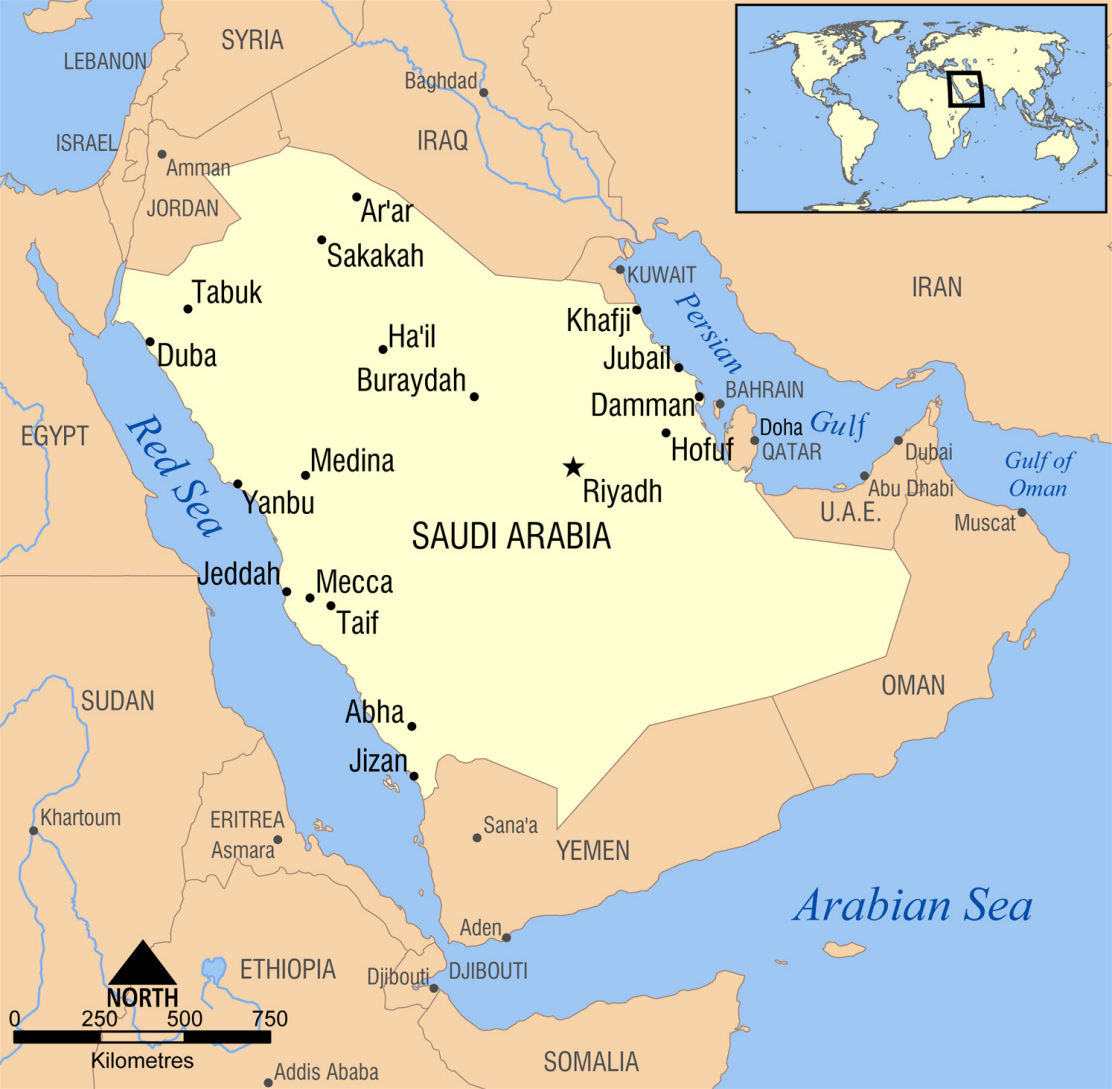
Map of Saudi Arabia (*Source*: Wikimedia).

Saudi Arabia (officially, Kingdom of Saudi Arabia) was established as a sovereign country in 1932, by its founder King Abdulaziz Al-Saud who led a successful campaign that lasted 30 years following the conquest of Riyadh (official capital) to unite large swathes of the Arabian Peninsula. With an area of 2,150,000 km^2^, Saudi Arabia occupies 80% of historical Arabia and is the 13th largest country in the world (Fig. 1). The King in Saudi Arabia also presides over the Council of Ministers, which represents the executive branch of Government although it also has the power to approve and veto legislations proposed by the Shura Council (Parliament), which has a largely advisory role.

The presence of the Islamic holiest sites in Mecca and Medina has endowed Saudi Arabia with a special status to the world's one billion Muslims since its establishment (the King of Saudi Arabia carries the official title of The Custodian of The Two Holy Mosques). With the discovery of massive oil reserves and other natural resources shortly after it was founded, Saudi Arabia has enjoyed vast revenues that made it possible to implement a very ambitious social and economic modernization strategy that transformed a mostly illiterate population to one with a literacy rate of 94.4% (98.1% for those <50 years of age who represent 75% of the population), and to become a member of G20, a forum of the world's 20 major economies.

## Population Structure

The population size in Saudi Arabia as of 2012, is 29.2 million, mostly Saudi nationals but with a significant minority (∼30%) of expatriates who come from many countries around the world, for example, there are >2.8 million Indians and around 1 million Pilipino. In addition to the ethnic Arabs who represent the overwhelming majority, there is a significant minority of Saudi nationals who largely descended from waves of immigrants who opted to stay in close proximity to the Holy Mosques in Mecca and Medina and were assimilated after the founding of modern Saudi Arabia. This minority represents a remarkably diverse group of ethnicities mostly from Asia and Africa. Virtually all Saudi nationals proclaim Islam as their religious affiliation. This has important implications when I discuss the ethical aspects surrounding genetics and genomic medicine in the country.

The relatively large size of Saudi families (average 6) has its roots in ancient Arabia when the large number of children was a source of pride. Large family size was further encouraged by Islam, the faith that was quickly embraced by nearly all inhabitants of Arabia shortly after its introduction >1400 years ago. Consanguinity is also an ancient practice that continues to be observed in more than half of the contemporary marriages in Saudi Arabia. Islam regulated this practice by proscribing a strict code that absolutely prohibits marriage between first and second degree relatives, but permits marriage between cousins. This permission is not equivalent to encouragement as some erroneously infer; Prophet Mohammed himself did marry women who were unrelated to him including one from a Jewish tribe. In addition to the commonly cited factor of “wealth preservation”, a powerful and yet less known mechanism that perpetuates the practice of consanguinity is the traditional view that marriage is the natural course of women such that families should arrange within themselves to leave no woman unmarried. Although there are no more recent published data, the rates from 2008 suggest no decline compared to the rates published two or more decades earlier, indicating the resistance of this social practice to the wave of modernization that has swept the country over that period of time (El-Mouzan et al. 2007; Warsy et al. 2014). It remains to be seen if the recently published declining rates of consanguinity in neighboring countries with very similar cultural norms will replicate in Saudi Arabia. What is obvious, however, is that consanguinity will remain a powerful factor in shaping the landscape of genetic disorders in Saudi Arabia for the foreseeable future.

## Health Services in Saudi Arabia

Saudis in general favor a strong contribution of Government to their life in return for its control over the country's vast natural resources. Just like education, which is offered freely from K12 to doctorate and postdoctorate degrees, health is also provided freely for all citizens. Expatriates are entitled to health insurance provided by their employers as mandated by law and receive their health care through an extensive network of private-run healthcare systems. The public healthcare system is mostly under the governance of the Ministry of Health (MOH) and consists of 2259 primary care centers, and 259 hospitals. The doctor/population ratio and hospital/population ratios at 24.4/10,000 and 20.7/10,000, respectively, are below that of many developed countries but newer plans have been revealed to improve this ratio. Law-enforcement personnel are entitled, in addition to MOH-run health care, to a large network of primary care centers and hospitals that are run by the Ministry of Interior. Similarly, military and National Guard personnel and their families enjoy the additional medical services that are administered by the respective agencies. The author's own institution (KFSHRC) is a general organization that is funded by the Government and offers highly specialized health care independent of MOH. The private sector consists of a vast network of private practices, usually in the form of polyclinics that fall under one administration, as well as secondary and tertiary hospitals. Although this sector represents the sole healthcare provider for noncitizens, many citizens also receive their healthcare in the private sector by choice, for example, to avoid a long wait-time in the public sector. This fragmentation of healthcare delivery has created a number of challenges towards the adoption of a national healthcare strategy equivalent to other countries with socialized medicine, for example, NHS in the UK. Mortality rate statistics are well below the global average but not yet on par with those of more developed countries. For example, mortality rate of children less than 5 is 12/1000 and maternal mortality is 7/100,000 births (global average is 44/1000 and 209.1/100,000, and Western Europe has an average of 3.9/1000 and 6.3/100,000, respectively) (Kassebaum et al. 2014; Wang et al. 2014). Life expectancy has also increased to 73.8 (compare to 80.3 years in Western Europe). This improvement in healthcare delivery has resulted in reduction in communicable diseases and brought noncommunicable diseases including genetic disorders to the forefront of national healthcare agenda.

## Genetic Services in Saudi Arabia

There is more than 30 board certified clinical geneticists in Saudi Arabia, the overwhelming majority of whom practice in State-funded tertiary centers. Most of these physicians have received their specialization in clinical genetics abroad but an accredited local fellowship program in medical genetics has been graduating practicing medical geneticists since its establishment a few years ago. These physicians cover the major disciplines of clinical genetics: dysmorphology, inborn errors of metabolism, prenatal and cancer genetics, and are supported by a limited number of certified genetic counselors. The overwhelming number of patients with neurocognitive phenotypes compels neurologists, especially pediatric neurologists, to frequently assume the role of a clinical geneticist since the average wait-time for clinical geneticists is often >8 months. Similarly, because thalassemias and hemoglobinopathies are the most frequent Mendelian diseases in Saudi Arabia (see below), hematologists usually take care of counseling these families and only refer the most atypical cases to clinical genetics for workup or counseling.

Cytogenetic testing is widely available, usually in the form of traditional karyotyping and FISH analysis. Molecular karyotyping is only available in a few centers. The major molecular diagnostic laboratory is at KFSHRC (Saudi Diagnostic Laboratory or SDL), which tests for 66 single gene disorders. We are currently validating the “Mendeliome” assay, which uses new multiplexing methods to amplify ∼3000 Mendelian genes known to cause human diseases followed by next-generation sequencing, on 3500 patients. Once validated, this test will be available for all patients with suspected genetic diseases as an intermediary test before considering whole-exome or whole-genome sequencing (details will be published elsewhere). Whole-exome and whole-genome sequencing are only available on research basis locally but SDL plans to launch these on clinical basis in the very near future.

The first Saudi national newborn screening program was for congenital hypothyroidism and was established in November 1989 (Al-Jurayyan et al. 1996). The pioneering work of the Tandem Spectrometry Lab at KFSHRC on the use of electrospray in the implementation of tandem spectrometry in the analysis of various metabolites in body fluids is noteworthy. It has set the stage for the first implementation of computer-assisted algorithm in the simultaneous estimation of many metabolites and flagging of abnormal results, the basis of today's newborn screening around the world (Rashed et al. 1994, 1995, 1997, 1999). Owing to this history, KFSHRC has a long tradition in performing newborn screening for 16 different inborn errors of metabolism, which evolved into a pilot program starting in 2004 to screen newborns from participating hospitals around the country. More recently, the MOH has assumed full responsibility of newborn screening, which is now performed as a national program. There are no national guidelines on newborn screening for deafness, which is left to the discretion of the individual hospitals.

While the newborn screening program was widely accepted, the premarital screening program was more controversial. After considerable deliberation, a law was passed in 2002 that mandates screening for hemoglobinopathies, thalassemias, and G6PDH deficiency prior to issuing a marriage contract. Aside from the controversy surrounding the issue of autonomy, the program delivered sobering results after its establishment with nearly 90% of “incompatible” couples moving ahead with their marriage plans (the law explicitly allows couples to exercise freedom of choice upon learning their results) (AlHamdan et al. 2007). This was clearly the result of inadequate pre- and posttest counseling. Indeed, major developments in the program to address these deficiencies have significantly reduced the percentage of “incompatible” marriages to a national average of 40%, with marked regional variations (large cities such as Riyadh are nearing 20% whereas rural areas with strong tribal traditions continue to see a majority of “incompatible” couples moving ahead with marriage) (Memish and Saeedi 2011) (Ayman Alsulaimani, pers. comm.). There is strong interest in expanding the premarital screening program to include all Mendelian disorders by utilizing the newly available and affordable next-generation sequencing tools, and local research is ongoing in order to provide empirical data on the practicality of this approach.

Prenatal genetics is largely practiced by maternal-fetal medicine specialists due to severe deficiency in the number of qualified clinical geneticists. Recent years have witnessed a tremendous growth in the demand for chorionic villous sampling and amniocentesis for the diagnosis of single gene disorders. At KFSHRC alone, the number of prenatal samples that are tested for single gene disorders has increased from 5 in 2004 to 250 in 2013. Therapeutic abortion is permitted by law if performed within 120 days from the time of fertilization in order to comply with the Islamic view of the timing of ensoulment (Alkuraya and Kilani 2001). However, the approved indication for the procedure, which is “severe malformation”, must be authorized by three attending-level physicians. The definition of “severe” is left to the discretion of the medical team after consulting with the family. For example, intellectual disability is a common indication for many therapeutic abortion procedures. Contrary to commonly held views, we have shown that early prenatal diagnosis is the method of choice for couples who had one or more children with single gene disorders, as long as they are provided with a culturally sensitive genetic counseling that addresses their religious and cultural concerns (Alkuraya and Kilani 2001). Nearly 45% of these couples opt for early prenatal diagnosis compared to 35% who choose preimplantation genetic diagnosis (PGD) (Alkuraya 2013a). PGD is available freely at KFSHRC but is also provided by the private sector. Noninvasive prenatal screening using cell-free fetal DNA in maternal blood is quickly becoming integrated in prenatal care. KFSHRC offers this test routinely to all pregnant women regardless of their perceived risk and the MOH is considering making this test available throughout its vast network of hospitals and medical centers.

## Genetic Disorders in Saudi Arabia

Not surprisingly, the high rate of consanguinity has greatly impacted the landscape of genetic disorders in Saudi Arabia and a quick search for published genetic diagnoses from Saudi Arabia readily reveals the clear bias toward autosomal recessive disorders. There are important practical implications of the role consanguinity plays in shaping the genetics of Mendelian diseases in Saudi Arabia. For recessive disorders, consanguinity favors homozygosity over compound heterozygosity, especially for less common conditions, and this is reflected in the finding that the overwhelming majority of recessive mutations identified in Saudi diagnostic laboratories are homozygous, a pattern that is echoed by published studies from Saudi Arabia (Alkuraya 2010a). This phenomenon can easily be leveraged in the area of diagnostics such that an inexpensive genome-wide homozygosity scan can greatly aid in the diagnostic work up as shown in detail elsewhere (Alkuraya 2010b). For example, examining the genes within the homozygous intervals can easily help the clinician to either confirm or reconsider an uncertain clinical diagnosis. This can also help guide the sequencing effort when a disorder is genetically heterogeneous, especially when the mutation is not readily detectable, for example, deep intronic, where prioritizing a particular gene can make more involved tests, for example, RTPCR, more justifiable. One could argue that this is less relevant now with the availability of whole-exome sequencing. However, a homozygosity scan can greatly reduce the number of candidate variants as we have shown in many instances (Alkuraya 2013b). That consanguinity can render homozygous DNA variants that arose as recently as two generations ago (in the case of first cousin union) makes it possible for private mutations to be overrepresented and for allelic heterogeneity to be common as we have shown previously (Aldahmesh et al. 2009). This has important implications, in that screening approaches that rely on common mutations are unlikely to be effective in Saudi Arabia, hence the push for sequencing-based approaches (Kaya et al. 2011). Interestingly, this level of homozygosity has the potential to reveal unusual patterns of inheritance. In addition to pseuododominance inheritance, which is seen not infrequently, classical dominant disorders may assume a recessive pattern of inheritance, for example, we have a case of Treacher-Collins syndrome caused by a homozygous truncating mutation in *TCOF1* while the heterozygous parents were completely unaffected (unpublished). Alternatively, the same gene that is known to cause a particular phenotype in the heterozygous state may result in a novel phenotype in the homozygous state as we have shown for *ELOVL4* (Aldahmesh et al. 2011a).

Similar to the practice of clinical genetics elsewhere, syndromic and nonsyndromic forms of intellectual disability and developmental delay account for the majority of referrals to pediatric genetic services in Saudi Arabia. Our unpublished data clearly show that the majority of these cases have an underlying recessive cause of their disability, which is in clear contrast to outbred populations where recent studies on the utility of whole-exome sequencing revealed little or no contribution of recessive mutations (de Ligt et al. 2012; Rauch et al. 2012).

Many disorders have been first described/mapped in Saudi patients (Table 1). Other disorders are known to exist elsewhere but are particularly common in Saudi Arabia (Table 2). For some, this can easily be explained by the disease's high degree of genetic heterogeneity such that consanguinity can be an important catalyst in unmasking the recessiveness of numerous potential mutations across many loci, for example, ciliopathies, retinal dystrophies, and deafness. For others, a strong founder effect can be invoked as in many inborn errors of metabolism (1.5 in 1000 newborns are diagnosed with a metabolic disease in the Saudi newborn program) and congenital glaucoma. Geographic variation in the incidence of diseases has been suggested by some but the mobility of the population lessens the practical utility of this map especially when one considers that the geographic variation falls largely along tribal lines, which suggests that knowledge about the tribal origin can be more helpful clinically (Al-Owain et al. 2012).

**Table 1:** Clinical conditions first described in Saudi Arabia

Condition	Gene	Reference
Arthrogryposis, Perthes disease, and upward gaze palsy	*?*	
Retinal dystrophy with severe white matter changes	*ACBD5*	Abu-Safieh et al. (2013)
Weill–Marchesani-like syndrome	*ADAMTS17*	Morales et al. (2009)
Microcornea, myopic chorioretinal atrophy, and telecanthus (MMCAT)	*ADAMTS18*	Aldahmesh et al. (2013b)
Intellectual disability-strabismus syndrome	*ADAT3*	Alazami et al. (2013)
*AGK*-related cataract	*AGK*	Aldahmesh et al. (2012a)
Hypopituitarism, microcephaly, and visual and renal anomalies	*ARNT2*	Webb et al. (2013)
*BRCA2*-related primordial dwarfism	*BRCA2*	Shaheen et al. (2014a)
Microphthalmia-dysgenesis of corpus callosum-epilepsy	*C12orf57*	Zahrani et al. (2013)
*C21orf2*-related retinal dystrophy	*C21orf2*	Abu-Safieh et al. (2013)
Woodhouse–Sakati syndrome	*C2orf37*	Alazami et al. (2008)
Cognitive impairment, dysmorphic facies and skeletal abnormalities syndrome	*CACNA1G*	Al-Owain et al. (2011)
*CENPJ*-related Seckel syndrome	*CENPJ*	Al-Dosari et al. (2010)
Intellectual disability-hypohidrosis syndrome	*COG6*	Shaheen et al. (2013a)
*COLEC11*-related Malpuech syndrome	*COLEC11*	Rooryck et al. (2011)
*CRIPT*-related primordial dwarfism	*CRIPT*	Shaheen et al. (2014a)
*CSPP1*-related Meckel–Gruber syndrome	*CSPP1*	Shaheen et al. (2014b)
Lethal familial hyperekplexia-brain malformation syndrome	*CTSD*	Seidahmed et al. (2012)
Myopia with dysmorphism	*CTSH*	Aldahmesh et al. (2013a)
*CYP51A1*-related cataract	*CYP51A1*	Aldahmesh et al. (2012b)
*DDX59*-related oral-facial-digital syndrome	*DDX59*	Shamseldin et al. (2013)
*DNA2*-related Seckel syndrome	*DNA2*	Shaheen et al. (2014a)
*DNASE1L3*-related SLE	*DNASE1L3*	Al-Mayouf et al. (2011)
*DOCK6*-related Adams-Oliver syndrome	*DOCK6*	Shaheen et al. (2011a)
Retinal dystrophy with myopathy	*DTHD1*	Abu-Safieh et al. (2013)
Ichthyosis, spastic quadriplegia, and mental retardation	*ELOVL4*	Aldahmesh et al. (2011a)
*EMC1*-related retinal dystrophy	*EMC1*	Abu-Safieh et al. (2013)
*EOGT*-related Adams-Oliver syndrome	*EOGT*	Shaheen et al. (2013b)
Pellagra-like syndrome	*ERCC5*	Hijazi et al. (2013)
*ERLIN2*-related complex hereditary spastic paraplegia	*ERLIN2*	Alazami et al. (2011)
*EVC2*-related Meckel–Gruber syndrome	*EVC2*	Shaheen et al. (2012a)
*FARS2*-related mitochondrial encephalomyopathy	*FARS2*	Shamseldin et al. (2012a)
*FBXL4*-related mitochondrial encephalomyopathy	*FBXL4*	Gai et al. (2013)
Bruck syndrome 1	*FKBP10*	Shaheen et al. (2010)
*G6PC3*-related cyclic neutropenia	*G6PC3*	Alangari et al. (2013)
*GPR125*-related retinal dystrophy	*GPR125*	Abu-Safieh et al. (2013)
*IFT27*-related Bardet–Biedl syndrome	*IFT27*	Aldahmesh et al. (2014)
Familial retinal artery macroaneurysm	*IGFBP7*	Abu-Safieh et al. (2011)
Congenital hyperinsulinemia with rhabdomyolysis	*KCNJ11*	Albaqumi et al. (2014)
*KIAA1549*-related retinal dystrophy	*KIAA1549*	Abu-Safieh et al. (2013)
*KLHL41*-related myopathy	*KLHL41*	Gupta et al. (2013)
Facial dysmorphism with severe growth deficiency	*LARP7*	Alazami et al. (2012)
*LRBA*-related Crohn's disease with immunodeficiency	*LRBA*	Alangari et al. (2012)
*LRPAP1*-related myopia	*LRPAP1*	Aldahmesh et al. (2013a)
*MEOX1*-related Klippel–Feil syndrome	*MEOX1*	Mohamed et al. (2013)
*METTL23*-related intellectual disability	*METTL23*	Reiff et al. (2014)
*MFF*-related mitochondrial encephalomyopathy	*MFF*	Shamseldin et al. (2012a)
*MMP2*-related multicentric osteolysis	*MMP2*	Al-Aqeel (2005)
*MPDZ*-related hydrocephalus	*MPDZ*	Al-Dosari et al. (2013)
*MRI1*-related infantile epilepsy with severe cystic degeneration of the brain	*MRI1*	Sunker and Alkuraya (2013)
Bone marrow failure with facial dysmorphsim	*MYSM1*	Alsultan et al. (2013)
*NECAP1*-related early infantile epileptic encephalopathy	*NECAP1*	Alazami et al. (2014a)
*ODZ3*-related microphthalmia	*ODZ3*	Aldahmesh et al. (2012c)
*OPLAH*-related oxoprolinurai	*OPLAH*	Almaghlouth et al. (2012)
*PHC1*-related microcephaly	*PHC1*	Awad et al. (2013)
*PHGDH*-related Neu-Laxova syndrome	*PHGDH*	Shaheen et al. (2014c)
*PITX3*-related microphthalmia	*PITX3*	Aldahmesh et al. (2011b)
*POC1A*-related primordial dwarfism	*POC1A*	Shaheen et al. (2012b)
*RAB33B*-related Smith–McCort dysplasia	*RAB33B*	Alshammari et al. (2012)
CMT-microcephaly-syndactyly-intellectual disability	*SBF1*	Alazami et al. (2014b))
*SCLT1*-related oral-facial-digital syndrome	*SCLT1*	Adly et al. (2014)
*SEC8*-related Meckel–Gruber syndrome	*SEC8*	Shaheen et al. (2012a)
*SIX6*-related autosomal recessive microphthalmia	*SIX6*	Aldahmesh et al. (2013c)
*TBC1D32*-related oral-facial-digital syndrome	*TBC1D32*	Adly et al. (2014)
Congenital hypoparathyroidism, severe growth failure, and dysmorphic facies	*TBCE*	Sanjad et al. (1991)
*TCTN2*-related Meckel–Gruber syndrome	*TCTN2*	Shaheen et al. (2011b)
*TMEM231*-related Meckel–Gruber syndrome	*TMEM231*	Shaheen et al. (2013c)
*TMEM38*-related osteogenesis imperfecta	*TMEM38B*	Shaheen et al. (2012c)
Osteogenesis imperfecta with profound neurological impairment	*WNT1*	Faqeih et al. (2013)
*XRCC2*-related Fanconi anemia	*XRCC2*	Shamseldin et al. (2012b)
*XRCC4*-related primordial dwarfism	*XRCC4*	Shaheen et al. (2014a)

**Table 2:** Frequently encountered Mendelian conditions in Saudi Arabia

Sickel-cell anemia
Thalassemia
Intellectual disability
Congenital glaucoma
Bardet–Biedl syndrome
Meckel–Gruber syndrome
Organic acidemias
Lysosomal storage disorders
Retinal dystrophies
Hearing loss
Primary microcephaly

## Opportunities in Genomic Medicine in Saudi Arabia

The high rate of consanguinity in Saudi Arabia has long been exploited to accelerate the annotation of recessive Mendelian genes and the recent years have witnessed a marked shift towards building infrastructure that permits this line of research to be performed locally. This trend has made a positive impact on the attitude of young Saudis to pursue careers in human genetics. But the study of rare recessive Mendelian disorders is only one of many opportunities that genomic research in Saudi Arabia has to offer. For example, identification of Mendelian forms of common diseases can provide novel insights into pathogenic mechanisms that could prove relevant to the common forms of these diseases (Al-Mayouf et al. 2011; Alangari et al. 2012; Aldahmesh et al. 2013a). Beyond Mendelian disorders, genomic analysis of Saudis has proved to be a valuable resource to track nullizygous DNA segments and biallelically inactivated genes in nondiseased individuals (Khalak et al. 2012). Not only does this line of research have the potential to improve the annotation of the human genome in terms of its clinical relevance, but it can also identify novel druggable targets by identifying genes whose loss of function brings about desirable phenotypic traits as recently shown with *PCSK9* and *CCR5* (Lederman et al. 2006; Rader and Daugherty 2008). In addition, the lack of representation of Saudi genomes in international GWAS consortia presents an opportunity to identify potentially novel risk alleles for common diseases as shown recently with the identification of a novel risk allele for complications of HBV infection (Al-Qahtani et al. 2013). A very recent study has shown the potential of genetically isolated societies to reveal novel risk alleles using a fraction of the usual study cohort size for a typical GWAS (Moltke et al. 2014), and this should provide an additional impetus to explore the genetics of common diseases among Saudis.

In recognition of these opportunities, the Saudi Government has recently announced its plan to fund the sequencing of 100,000 Saudis as part of the newly launched Saudi Human Genome Project. The above lines of research and others will form the basis of selecting the 100,000 Saudis to be sequenced. For example, 10,000 healthy Saudis will have their genomes sequenced specifically in search of biallelically inactivated genes (Kaiser 2014).

It is clear that Saudi Arabia has been and will continue to be an important resource in the study of Mendelian genes, and recent technological advances are diversifying the relevance of this resource to the various fields of genomic medicine. The time has never been more opportune for conducting genomic research in Saudi Arabia to empower Saudis to reap its promise of better health.
